# Salivary microbial profiles in relation to age, periodontal, and systemic diseases

**DOI:** 10.1371/journal.pone.0189374

**Published:** 2018-03-14

**Authors:** Ronaldo Lira-Junior, Sigvard Åkerman, Björn Klinge, Elisabeth A. Boström, Anders Gustafsson

**Affiliations:** 1 Karolinska Institutet, Department of Dental Medicine, Division of Oral Diseases, Stockholm, Sweden; 2 Rio de Janeiro State University, Faculty of Odontology, Department of Periodontology, Rio de Janeiro, Brazil; 3 Malmö University, Faculty of Odontology, Department of Orofacial Pain and Jaw Function, Malmö, Sweden; 4 Malmö University, Faculty of Odontology, Department of Periodontology, Malmö, Sweden; Kanagawa Dental University, JAPAN

## Abstract

**Background:**

Analysis of saliva is emerging as a promising tool to diagnose and monitor diseases which makes determination of the salivary microbial profile in different scenarios essential.

**Objective:**

To evaluate the effects of age, periodontal disease, sex, smoking, and medical conditions on the salivary microbial profile.

**Design:**

A randomly selected sample of 441 individuals was enrolled (51% women; mean age 48.5±16.8). Participants answered a health questionnaire and underwent an oral examination. Stimulated saliva was collected and the counts of 41 bacteria were determined by checkerboard DNA-DNA hybridization.

**Results:**

Elderly participants (> 64 years old) presented a significant increase in 24 out of 41 bacterial species compared to adults (≤ 64 years old). *Eubacterium nodatum*, *Porphyromonas gingivalis*, and *Tannerella forsythia* were significantly higher in participants with generalized bone loss compared to without. Males and non-smokers had higher bacteria counts in saliva. Individuals having mental disorders or muscle and joint diseases showed significantly altered microbial profiles whereas small or no differences were found for subjects with high blood pressure, heart disease, previous heart surgery, bowel disease, tumors, or diabetes.

**Conclusion:**

Age, periodontal status, sex, smoking, and certain medical conditions namely, mental disorders and muscle and joint diseases, might affect the microbial profile in saliva.

## Introduction

The use of saliva as an attractive tool to investigate and monitor pathological conditions in the human body is increasingly frequent. Most compounds found in serum can also be found in saliva which makes saliva reflect the physiological state of the body. Saliva offers the advantage of its collection being easy and non-invasive [1]. Research on saliva has mainly been conducted to investigate inflammatory markers in relation to different oral and systemic conditions [2–5], whereas little attention has been devoted to assess how different medical conditions are associated with changes in the salivary microbiota.

The oral microbiota is complex with more than 700 species identified [6,7]. Salivary microbiota is dominated by the *Firmicutes* phylum and in health, the bacterial composition resembles the microbiota of the tongue, tonsils, and throat [8]. Saliva supplies the resident oral microbiota with nutrients, as well as antimicrobial factors that contribute to the regulation of the resident microbiotaand maintenance of the microbial homeostasis [9].

The presence of oral diseases, such as periodontitis and caries, have been associated with an altered salivary bacterial profile [10,11]. Also, lifestyle factors such as smoking [12], as well as systemic conditions, for instance inflammatory bowel disease, have been associated with changes in the oral microbiota [13] however, the specificity of the alterations in the microbiota has not been addressed in detail. Environmental conditions specific to the host’s systemic health, diet, genetic predisposition, and salivary antimicrobials are believed to shape the microbial community [9,14]. It is becoming evident that the microbiome plays a role in physiology and immunity and can be viewed as a forgotten “organ” [15] which highlights the importance of evaluating also the salivary microbiota profile in different settings.

Aging is associated to changes in virtually all organs. In the oral cavity, changes with aging resemble those happening throughout the body, such as loss of muscle tone and degradation of hard and soft tissues [16]. The saliva composition also changes with aging, as evidenced by reduced salivary flow [17,18], and an increase in several inflammatory mediators such as matrix metalloproteinase (MMP)-8, a neutrophil collagenase and a surrogate marker of connective tissue destruction [18]. Although differences with aging have been documented in several physiological and pathological processes, little is known of the effects of aging on the bacterial salivary profile. Human gut microbiota is greatly affected by the aging process, as evidenced by changes in the proportion of *Firmicutes* and an enrichment of facultative anaerobes. This disturbed microbiota is associated with a higher inflammatory status [19]. It has been suggested that aging does not substantially affect the composition of the subgingival microbiota [20] however, the phylogenetic microbial structure varies with aging in other oral niches, including saliva [21].

As several factors, either resulting from the host or from the environment, may affect the bacterial profile in saliva [22], a characterization of the salivary microbiota in different scenarios is relevant. Therefore, this study aimed to assess the effects of age, periodontal disease, sex, smoking, and a range of medical conditions on the salivary microbiota.

## Material and methods

### Study population

One thousand individuals aged 20 to 89 years old, living in Skåne, a county in the southern part of Sweden, were randomly selected and invited to participate in the study. From the total invited, 966 individuals composed the initial sample, of which 451 individuals were examined clinically. The reasons for not taking part of the study have been described previously [23]. Briefly, the participants who neither participated in the clinical examination nor answered the questionnaire, were those who could not be reached by telephone or letter, were unable to participate in the study due to bad health or old age, or were simply not interested in participating in this study. The clinical examinations were performed between March 2007 and November 2008. Study protocol was approved by the Ethical Board at the Lund University, Sweden. Patients were informed and gave a written consent.

### Questionnaire

All participants clinically examined were requested to respond to a questionnaire, which one individual failed to answer. The questions concerned patient perception of oral health, oral healthcare needs, pain, use of oral healthcare, dental materials, and background factors [24]. Data collected also included presence of diseases, use of medication, smoking, and snuffing habits. A non-response analysis was performed with 175 individuals who were not able to or were not interested in being clinically examined. These participants were more likely to be born in Sweden and to have a lower educational level, and were missing a higher number of teeth than those participating in the study. Smoking was recorded as smokers or non-smokers. Participants were classified according to their age into adults (subjects ≤ 64 years old) and elderlies (subjects > 64 years old) [20,25]. The following medical conditions were recorded: heart disease (n = 35), previous heart surgery (n = 11), hypertension (n = 76), diabetes (n = 16), muscle and joint diseases (n = 102), bowel diseases (n = 31), tumors (n = 16), and mental illness (n = 26). All these variables were registered dichotomously as presence or absence.

### Clinical and radiographic examinations

Four dentists from the Department of Oral Diagnostics, Faculty of Odontology, Malmö University, performed 90.5% of the clinical examinations. The dentists were coordinated regarding the diagnostic criteria through comprehensive written instructions, practice, and through discussion of clinical cases. Periodontal clinical measurements were recorded at four sites on each tooth. Dental implants were excluded from all measurements. Presence of visible plaque was recorded after drying with air. Probing depth (PD) was measured with a standard periodontal probe (Hu-Friedy, IL, USA). The deepest pocket greater than 4 mm was registered. Bleeding on probing (BoP) was recorded after probing the pockets. Also, digital panoramic and bitewing radiographs (picturing from the distal surface of the canine to the mesial surface of the last molar) were taken. After that, eight individuals were excluded: two participants were edentulous, one was edentulous but with dental implants, four individuals had missing radiographs, and one individual had radiographs of unacceptable quality. Thus, the study sample comprised 443 participants (45.8% of the initial sample).

All subjects were classified according to their extent of marginal bone loss. A tooth as a whole was considered as having loss of supportive bone if mesial or distal surface was found to have bone loss. Therefore, three groups were defined: PD- group (n = 303): horizontal bone loss < 1/3 of the root length; PD group (n = 89): horizontal bone loss ≥ 1/3 of the root length in < 30% of the sites; and PD+ group (n = 49): horizontal bone loss ≥ 1/3 of the root length in ≥ 30% of the sites [5]. This analysis was performed by an independent examiner. Intra-examiner agreement was calculated by evaluating the horizontal bone loss in 100 individuals twice. Radiographs were randomly chosen to encompass an even distribution of all three different periodontal categories.

### Saliva collection

Stimulated saliva was collected during 5-minute chewing on 0.5 g of paraffin into a graded tube. Saliva volume was determined, excluding the foam, and the secretion rate per minute was recorded. Trained dental assistants performed the sampling. After collection, samples were immediately frozen at -20°C until processing. After centrifugation (500 g for 10 minutes at 4°C), supernatants were aliquoted and stored at -80°C. Each saliva aliquot was used only once for each analysis. This study included 441 saliva samples.

Salivary levels of interleukin (IL)-1β, IL-6, IL-8, tumor necrosis factor (TNF)-α, MMP-8, tissue inhibitor of metalloproteinases (TIMP)-1, and lysozyme were measured using Luminex, IFMA or ELISA assays and described previously [4,5].

### Microbial profile analysis

Salivary microbial profile was determined by checkboard DNA-DNA hybridization, including a panel of 41 bacterial species as previously described [26]. Mean count (x10^5^ cell) of each bacterial species was calculated. The microbial panel analysed in the study is described in the S1 File.

### Statistical analysis

Data analyses were performed using Statistical Package for Social Sciences (SPSS, version 20, IBM, USA). Continuous variables are presented as mean (±standard deviation), and categorical variables as frequencies. Student’s t-test, X^2^ test or One-Way ANOVA (with Bonferroni post-test) were used to compare the significance of differences in clinical, inflammatory, and microbial variables between/among groups whenever appropriate. Analysis of covariance was performed to assess the possible influence of age on the associations with the microbial profile. Pearson correlation coefficient was calculated to assess the correlations among age, periodontal parameters, inflammatory biomarkers, and the microbial profile. Statistical significance was set at 0.05. A reference group (n = 241) was derived to address the possible confounding factor of comparing the microbial profile of patients with a particular systemic disease to a group of patients having other systemic disease. This group was composed of participants free of any of the systemic conditions evaluated.

## Results

The effect of age, periodontal disease, sex, smoking status, and of different medical conditions (heart disease, previous heart surgery, hypertension, diabetes, bowel diseases, muscle and joint diseases, tumors, and mental illness) on the salivary microbial profile was analysed in 441 participants. The general characteristics of these subjects have been previously described [4,23].

### Microbial profile and inflammatory status in relation to age

**Fig 1:**
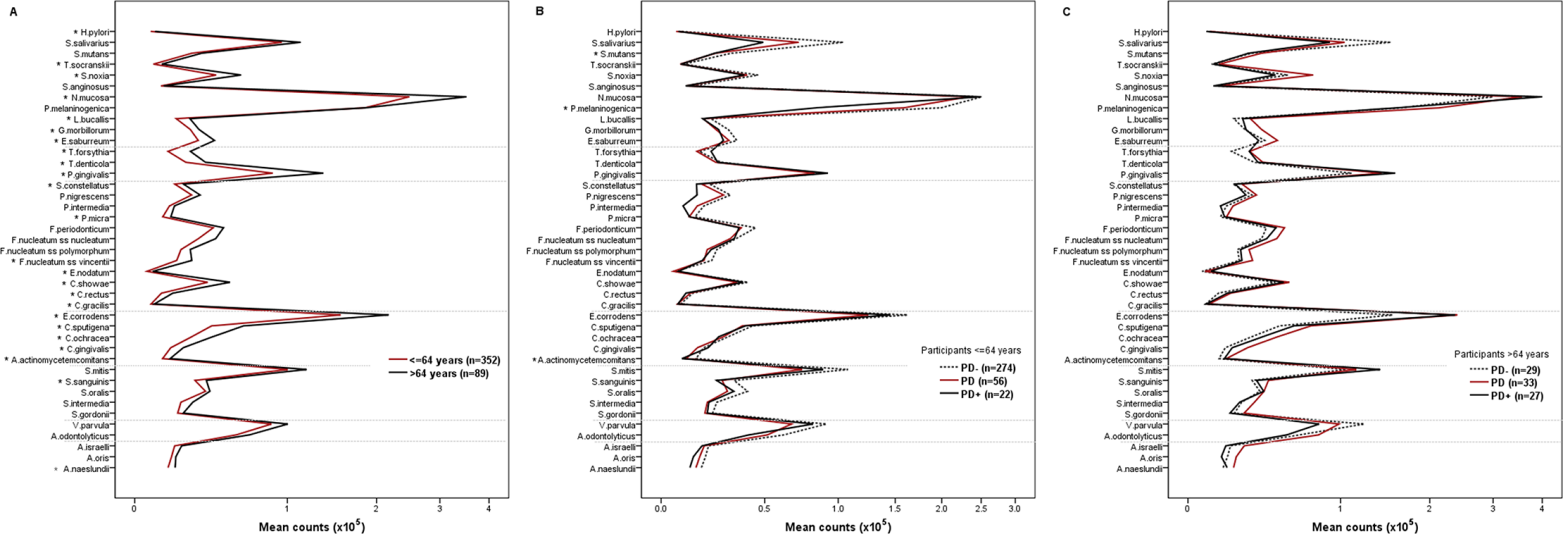
**Salivary mean counts (x10**^**5**^**) of 41 bacterial species in (a) participants ≤ 64 years old and participants > 64 years old and (b, c) according to their periodontal status.** *Significantly different between groups (Student’s t test or ANOVA with Bonferroni post-test).

We stratified this cohort according to age into adults (≤ 64 years old) and elderlies (> 64 years old) and assessed the relation between age and the salivary microbial profile. Elderly participants (n = 89) presented significantly elevated counts of 24 out of 41 species as compared to adults (n = 352) (Fig 1A). To further explore the microbial profile in adult and elderly participants, we analysed their microbiota according to the periodontal status. In this cohort, participants were classified as having no alveolar bone loss exceeding 1/3 of the root length (PD- group), localized alveolar bone loss (PD group), and generalized alveolar bone loss (PD+ group). In the adult group, significant differences were found for *A*. *actinomycetemcomitans*, *S*. *mutans*, and *P*. *melaninogenica* however, they lost significance in the post-hoc analysis (Fig 1B). In the elderly group, no significant differences were found in the microbiota among participants without periodontitis (PD-, n = 29), with localized periodontitis (PD, n = 33), and generalized periodontitis (PD+, n = 27) (Fig 1C).

When we explored the characteristics and periodontal parameters in adult and elderly participants, we found that the elderly group had fewer smokers, higher percentages of patients with medical disorders (diabetes, bowel disease, heart disease, hypertension, muscle and joint diseases, and tumors) and a worse periodontal condition. Regarding the salivary inflammatory markers IL-1β, IL-6, IL-8, TNF-α, MMP-8, TIMP-1, and lysozyme previously described in this cohort [4,5], the elderly group presented higher levels of IL-1β, IL-8, MMP-8, and MMP-8/TIMP-1 ratio and lower lysozyme levels than the adult group (Table 1).

**Table 1:** Characteristics, periodontal parameters and inflammatory markers according to age group (n = 441).

Variable	Adults (n = 352)	Elderlies (n = 89)	p-value*
Sex, male/female	172/180	43/46	NS
Smokers, n (%)	69 (19.6)	5 (5.6)	<0.01
Diabetes, n (%)	7 (2.0)	9 (10.2)	<0.01
Bowel disease, n (%)	18 (5.1)	13 (14.8)	<0.01
Heart disease, n (%)	12 (3.4)	23 (26.1)	<0.01
Hypertension, n (%)	36 (10.2)	40 (45.5)	<0.01
Muscle and joint diseases, n (%)	71 (20.2)	31 (35.2)	<0.01
Tumor, n (%)	6 (1.7)	10 (11.4)	<0.01
Mental illness, n (%)	20 (5.7)	6 (6.8)	NS
N° of teeth	26.53 (±2.60)	21.66 (±6.07)	<0.01
Plaque index	19.73 (±19.41)	31.33 (±27.85)	<0.01
Bleeding on probing	27.85 (±19.53)	32.10 (±24.47)	NS
% PD 4–5 mm	6.41 (±8.49)	10.26 (±11.24)	<0.01
% PD > 5 mm	0.53 (±2.12)	1.49 (±3.09)	<0.01
Salivary flow rate (ml/min)	1.54 (±0.75)	1.61 (±0.76)	NS
IL-1β (pg/ml)	64.02 (±98.15)	115.31 (±165.13)	<0.01
IL-6 (pg/ml)	8.00 (±11.90)	7.24 (±9.04)	NS
IL-8 (pg/ml)	421.73 (±476.57)	825.89 (±1216.12)	<0.01
MMP-8 (ng/ml)	267.67 (±223.42)	388.76 (±374.78)	<0.01
TIMP-1 (ng/ml)	266.16 (±190.59)	251.75 (±207.13)	NS
MMP-8/TIMP-1 ratio	0.45 (±0.50)	0.96 (±1.56)	<0.01
Lysozyme (ng/ml)	415.05 (±460.52)	316.94 (±232.58)	<0.01
Total protein concentration (μg/ml)	783.72 (±374.68)	969.22 (±571.75)	<0.01

Data presented as mean (±sd) or frequencies. PD: probing depth. NS: non-significant.

*Student’s t-test or Chi-square test.

Adults: participants aged ≤ 64 years. Elderlies: participants aged > 64 years.

**Fig 2:**
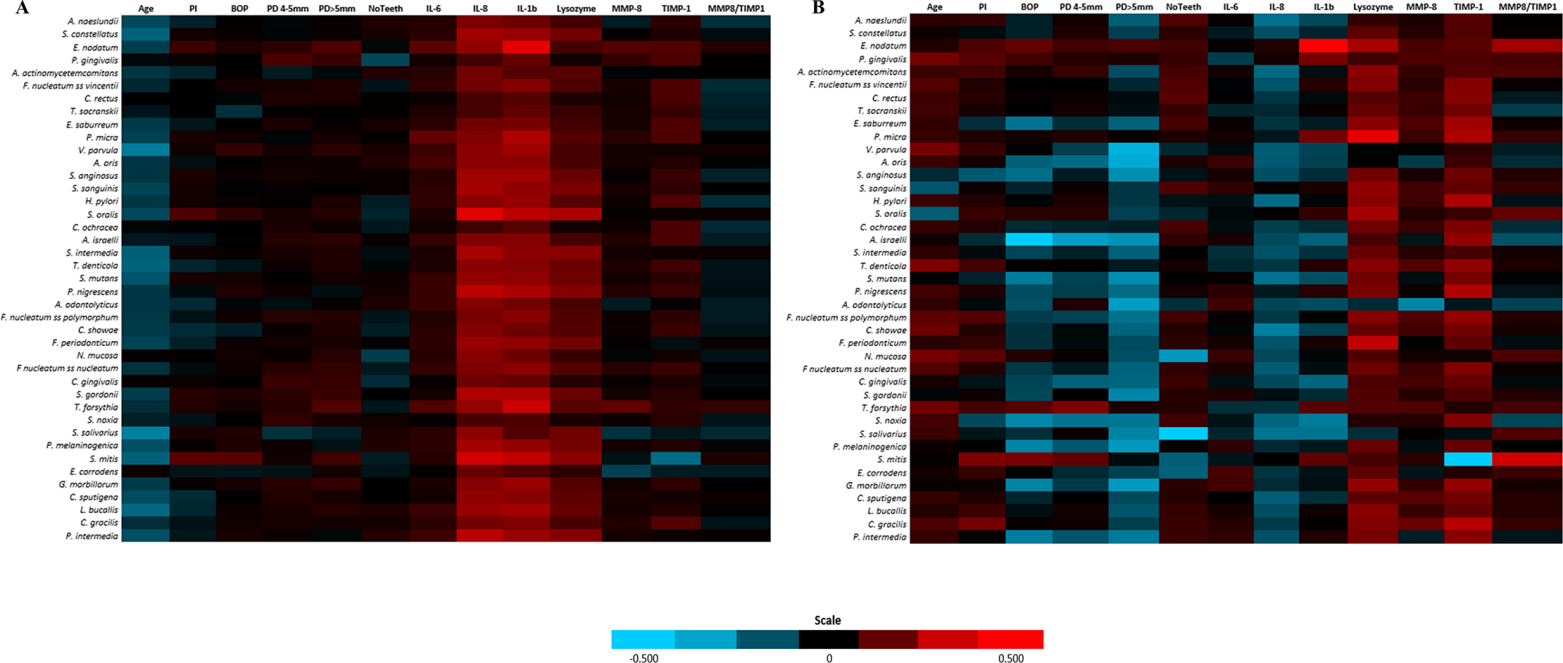
Correlation heatmap of age, periodontal parameters and inflammatory biomarkers with the salivary microorganisms in (a) participants ≤64 years old and (b) participants >64 years old.

We analysed the correlations among age, periodontal parameters (plaque index, BoP, PD 4–5 mm, PD > 5 mm, and number of teeth), and inflammatory markers with the salivary bacteria in adults (Fig 2A) and elderly participants (Fig 2B). In adults, IL-1β showed a moderate positive correlation with *E*. *nodatum* (r = 0.456) and *T*. *forsythia* (r = 0.406), and IL-8 with *S*. *oralis* (r = 0.444) and *S*. *mitis* (r = 0.412). In elderlies, IL-1β showed a moderate positive correlation with *E*. *nodatum* (r = 0.513), lysozyme with *P*. *micra* (r = 0.457), and the MMP-8/TIMP ratio with *S*. *mitis* (r = 0.409).

### Effects of periodontal status, sex, and smoking on salivary microbiota

**Fig 3:**
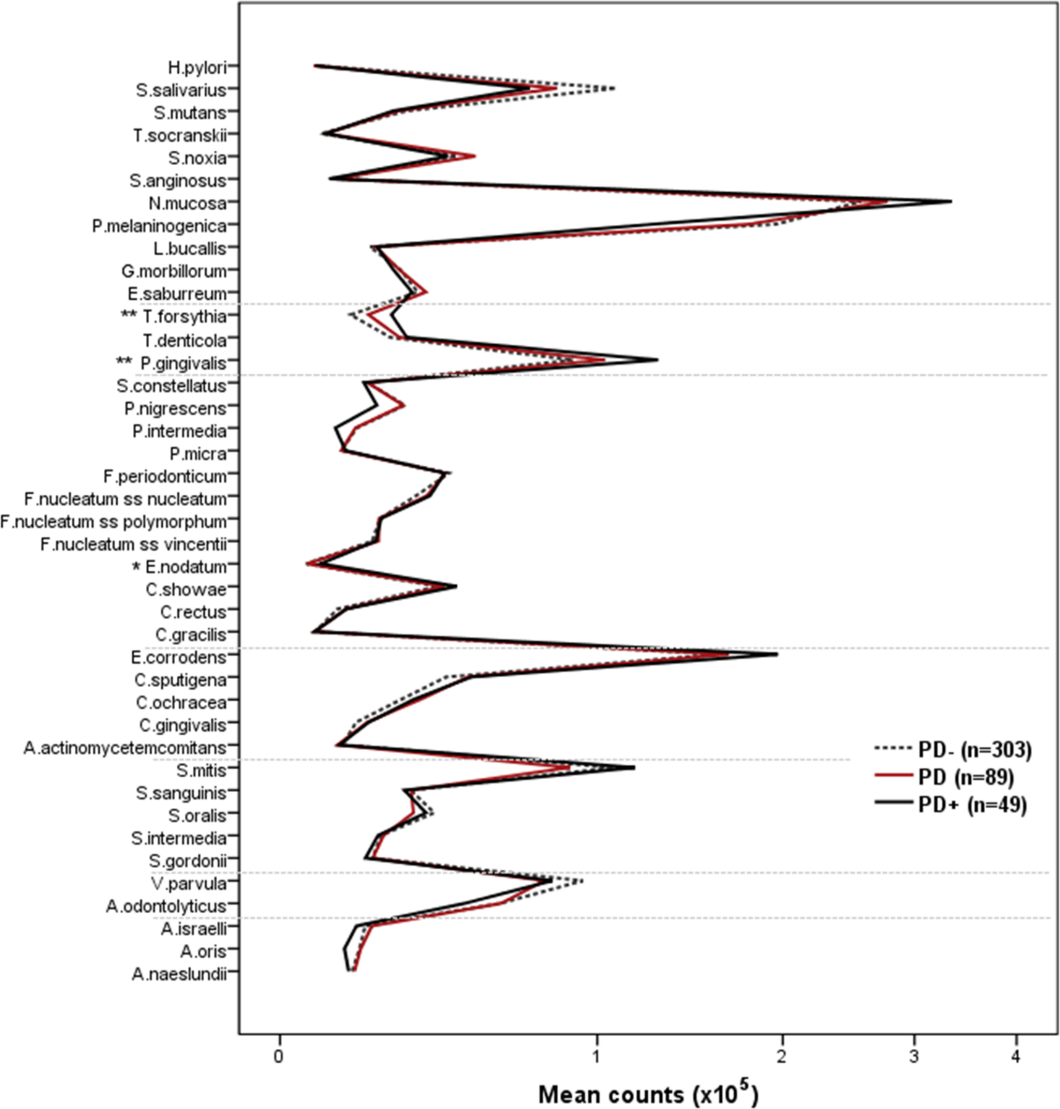
Salivary mean counts (x10^5^) of 41 bacterial species in participants with different periodontal status. *PD+ significantly different from PD- and PD groups. **PD+ significantly different from PD- group (ANOVA with Bonferroni post-test).

Regarding the association between periodontal status and the microbiota, significantly higher counts of *E*. *nodatum*, *P*. *gingivalis*, and *T*. *forsythia* were found in PD+ group (Fig 3). When restricted to non-smokers (n = 366), *E*. *nodatum*, *P*. *gingivalis*, *T*. *forsythia*, and *N*. *mucosa* were significantly higher in the PD+ group.

**Fig 4:**
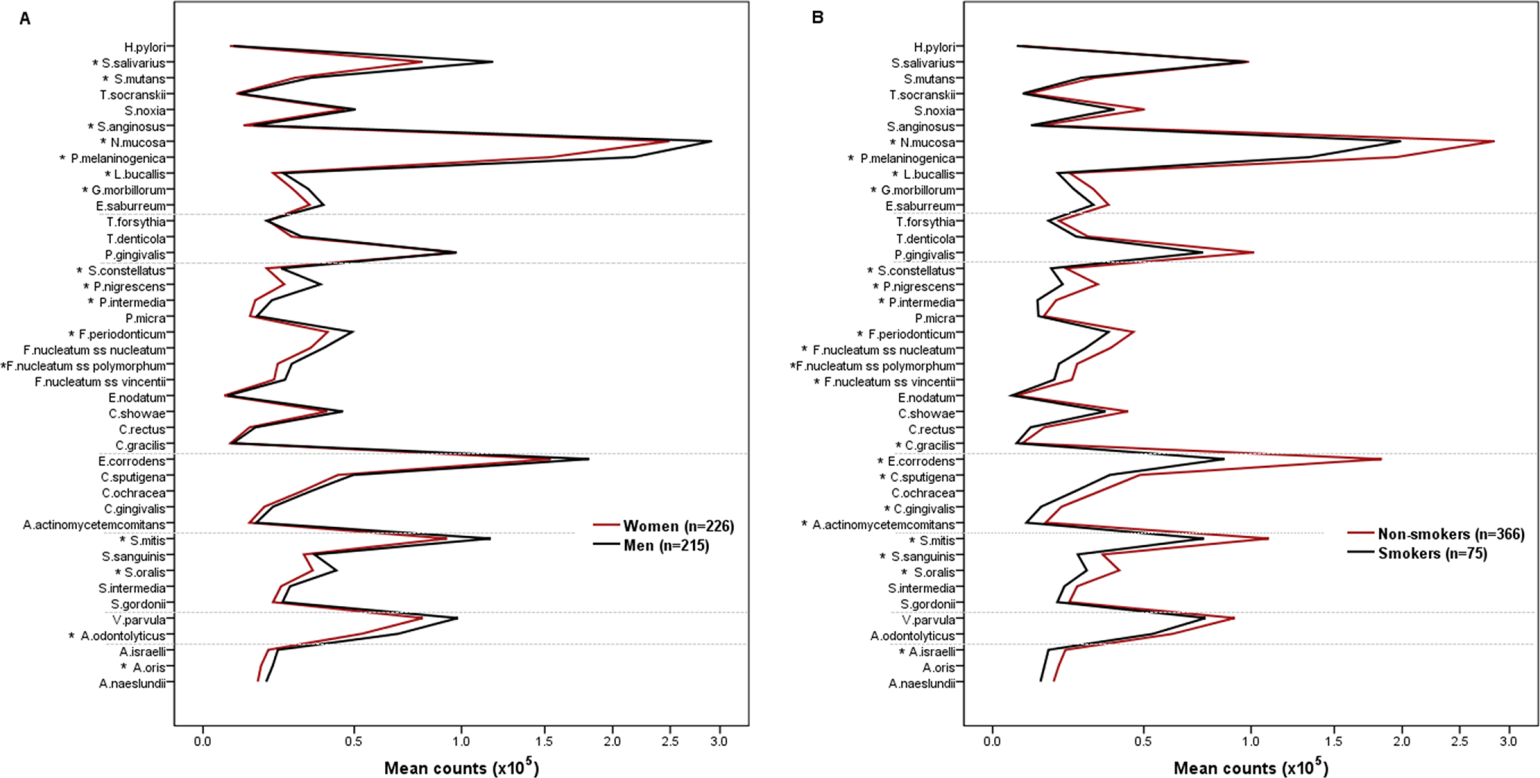
**Salivary mean counts (x10**^**5**^**) of 41 bacterial species according to (a) sex and (b) smoking status**. *Significantly different between groups (Student’s t test).

Concerning sex, men (n = 215) presented increased mean counts of 16 bacteria than women (n = 226) (Fig 4A). Regarding periodontal parameters, plaque index, BoP and PD 4–5 mm were significantly higher in men. When we stratified males and females by PD categories, there was no significant difference in the microbial profile among the three PD groups in males however, *E*. *nodatum* and *T*. *forsythia* counts were significantly higher in females belonging to the PD+ group.

When we analysed the influence of smoking, smokers (n = 75) exhibited significantly lower counts of 19 bacteria than non-smokers (n = 366) (Fig 4B). Sex, periodontal parameters, and the frequency of the PD groups did not differ significantly between smokers and non-smokers. Regarding medical status, mental disorders were more prevalent in smokers. Smokers were significantly younger than non-smokers. Therefore, we performed an analysis controlling for age and found that *S*. *constellatus*, *F*. *nucleatum ss vicentii*, *C*. *sputigena*, and *L*. *bucallis* lost significance, while *A*. *naeslundii* and *N*. *mucosa* became significantly different between groups.

### Influence of medical conditions on salivary microbiota

**Fig 5:**
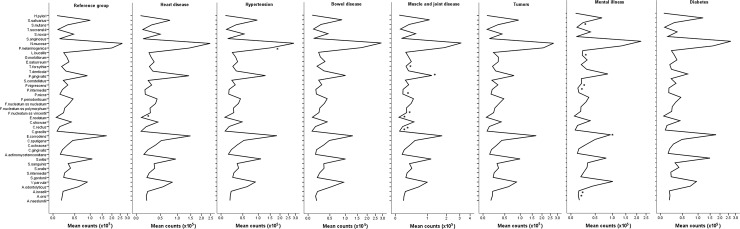
Salivary microbial profile in the reference group and in each systemic condition. *Significantly different in comparison to the reference group (Student’s t test).

We compared the microbiota of patients with each systemic disease to the reference group (n = 241) and the results are shown in Fig 5. Patients reporting heart disease (n = 35) presented significantly higher mean counts of *E*. *nodatum*. Patients with high blood pressure (n = 76) presented significantly increased mean counts of *P*. *melaninogenica*. Patients having mental illness (n = 26) exhibited lower counts of *A*. *oris*, *A*. *israelli*, *S*. *mutans*, *E*. *corrodens*, *G*. *morbillorum*, *P*. *nigrescens*, and *P*. *intermedia*. When we assessed the impact of muscle and joint diseases (MJD) on the microbial profile, patients having such conditions (n = 102) showed increased counts of 7 bacterial species. No significant differences in the salivary microbiota were found for subjects having bowel disease (n = 31), tumors (n = 16), or diabetes (n = 16).

To further assess the influence of periodontitis on the microbial profile of hypertensive and MJD participants, we stratified them according to their periodontal status (PD-, PD, and PD+). Hypertensive participants with generalized bone loss (PD+, n = 17) showed significantly higher counts of *S*. *mitis* than participants without (PD-, n = 33). MJD participants with generalized periodontitis (PD+ group) showed increased counts of *E*. *nodatum* in comparison with both patients without (PD- group) and patients with localized bone loss (PD group). *P*. *gingivalis*, *T*. *forsythia*, and *S*. *mitis* were increased in PD+ individuals in comparison with PD- participants. We did not stratify participants with other systemic diseases by their periodontal status due to low numbers per group in this cohort.

## Discussion

This study assessed the influence of different host and environmental variables on the microbial profile in saliva from a large group of participants. Alterations in the microbial profile and the inflammatory status in adults and elderlies were also evaluated. We found that aging was associated with increased counts of several bacteria in saliva, including the so-called periodontopathogens *A*. *actinomycetemcomitans*, *P*. *gingivalis*, *T*. *denticola*, and *T*. *forsythia*. Along with altered microbial profile, aging was also associated with increased inflammatory biomarkers in saliva.

In accordance with our results, age-related alterations have previously been reported in salivary microbiota [21,22,27]. Takeshita *et al*. [22] have found that age is significantly associated with changes in the salivary microbiome. On the opposite side, Belstrøm *et al*. [12] reported no significant difference in the presence or level of any taxon/cluster between age groups. Darout *et al*. [28] also did not find significant differences in the salivary levels of 25 bacteria between age groups (19–39 and ≥ 40 years old). However, it is important to underscore the differences in the definitions of the age groups among studies as it might have an impact on the results. Changes in oral microbiota are reported to be detected particularly after the age of 70 years [27]. Since we observed higher plaque index and increased percentage of periodontal pockets, we explored whether the increased microbial counts in elderlies could be due to a worse periodontal condition, but we found no significant difference in bacterial levels among periodontal groups in elderlies.

We reported higher levels of IL-1β, IL-8, MMP-8, and MMP-8/TIMP-1 ratio in elderlies, as well as lower lysozyme levels. In addition, moderate positive correlations were noted between some inflammatory markers and microorganisms in saliva from elderly participants. In agreement with our findings, higher levels of MMP-8 have been found in saliva of elderly individuals and mean levels of other salivary markers including prostaglandin E2 and MMP-9 activity are increased in aged subjects [18,29]. Aging has also been shown to impact inflammatory markers in blood, as evidenced by increased levels of IL-1β, IL-6, TNF-α in patients > 60 years old [30]. It can be noted that there was a correlation between *E*. *nodatum* and IL-1β in both age groups. *E*. *nodatum* is strongly associated with periodontitis and has been suggested to be included in the red complex in supragingival biofilm [31]. Correlations between salivary microorganisms and inflammatory markers in saliva have also been reported in patients with inflammatory bowel disease [13].

As expected, participants with generalized alveolar bone loss (PD+ group) presented higher mean counts of some members of the orange and red complexes, both in the whole cohort and in non-smokers. This is in line with previous studies reporting differences in salivary periodontal pathogens in periodontitis patients [10,32]. Other differences have also been reported in the salivary microbial profile of periodontitis patients, such as increased frequencies and higher levels of the putative periodontal pathogens *T*. *forsythia*, *P*. *micra*, and *F*. *alocis* [10]. We also found that male participants showed higher counts of several bacteria species in saliva compared to females. We speculate that this result could be due to a slightly worse periodontal condition in males than in females observed here. Belstrøm *et al*. [12] have found no differential effect of sex on salivary microbiota. On the opposite, higher levels of *A*. *israelii*, *C*. *gracilis*, and *P*. *gingivalis* have been found in females [28]. Yet, we found increased counts of *E*. *nodatum* and *T*. *forsythia* in female participants having generalized bone loss, but not in male participants. Paju *et al*. [32] have reported an association between number of pathogens and number of teeth with pockets ≥ 4 mm in women who had never smoked, and such association was not seen in men.

We observed an increase in several bacterial species in non-smoking individuals as compared to smokers. There are contradicting results regarding the influence of smoking on the salivary microbiota. Belstrøm *et al*. [12] have found higher levels of 2 bacterial taxa in saliva of smokers, while Mager *et al*. [33] have shown no significant elevation in 40 species in smokers, instead an elevation of *N*. *mucosa* was seen in saliva of periodontally healthy, non-smokers patients. In our cohort, non-smokers were significantly older than smokers, but most differences remained after compensation for age. Since we also reported a significant increase in bacteria counts in elderlies, this might have confounded the association between the microbial profile and both aging and smoking. Therefore, we also compared the microbiota between smokers and non-smokers only in adult subjects (≤ 64 years old). Non-smoking participants still showed increased counts of 15 out of 41 bacterial species (data not shown), thus suggesting the existence of a true effect of smoking in depressing salivary microbiota, since periodontal parameters were similar between both groups. The effects of smoking on salivary microbiota, specially using next-generation sequencing techniques, deserve further investigations.

The influence of several self-reported medical conditions on the salivary microbial profile was also evaluated in the present study. Subjects having bowel disease, tumors, diabetes, or previous heart surgery showed no significant alterations in salivary microbiota. Alterations in salivary microbiota have previously been shown in medical conditions, such as increase in the genus *Prevotella* in patients with inflammatory bowel disease [13], and decreased *N*. *elongata* and *S*. *mitis* in patients with pancreatic cancer [34]. We found that hypertensive patients showed a slight change in the salivary microbiota, as observed by higher counts of *P*. *melaninogenica*. However, patients with mental disorders and muscle and joint diseases showed more dramatic changes. Patients with mental disorders presented decreased counts of 7 bacteria. Disruption of the gastrointestinal microbiota might affect brain function through several pathways, and thus, has been implicated in mental disorders [35]. Salivary dysbiosis could be an alternative tool to further investigate the connection between mental diseases and microbiota.

We found significant increases in several bacterial species in patients having muscle and joint diseases, and increases were mainly found in bacteria belonging to orange complex. Also, patients having muscle and joint diseases and generalized bone loss (PD+ group) exhibited altered microbial profile in comparison with participants without. Altered salivary microbiome has been observed in patients with rheumatoid arthritis, which was partially resolved after treatment with disease-modifying antirheumatic drugs [36]. Salivary microbial changes are also seen in patients with Sjögren’s syndrome [37,38]. Siddiqui *et al*. [38] have shown a higher frequency of *Firmicutes* and decreased frequencies of *Synergistetes* and *Spirochaetes* in Sjögren’s syndrome patients.

The mechanisms and the implications behind alterations in bacterial profiles associated to systemic diseases are not clear, as well as how specific these changes are for each disease. One can argue that it might reflect an altered inflammatory profile, which would propitiate a better environment for growth of some microorganisms. In return, the dysbiotic microbiota would foster an inflammatory reaction. As such, Bajaj *et al*. [39] have found a microbial dysbiosis in saliva of patients with cirrhosis, and this dysbiosis was associated with inflammation, changes in bacterial defenses, and subsequent hospitalizations. The authors argued that the oral microbial changes are likely to follow a systemic pro-inflammatory milieu, which in turn is associated with worst outcomes.

This study has some limitations that must be taken into account when interpreting its findings. Firstly, this is a cross-sectional study and, therefore, no causal inference can be done. Large cohort studies are needed to determine temporality and assess the impact of the microbial changes on the diseases. Second, medical conditions were ascribed based on self-report and no verification was done from the anamnestic data, which implies a risk for undiagnosed diseases [4]. We also cannot exclude a possible impact of current medications on the results. Also, we did not specify which disease we were dealing with, rather diseases were grouped together in broader categories, for example muscle and joint diseases. Different diseases might have different influences on the salivary microbiota. Nevertheless, we believe it is a good approach to generate hypotheses from the results of this large group of individuals. Third, we analysed 41 bacterial species by checkboard DNA-DNA hybridization and the salivary microbiota is more complex. Therefore, further or new differences in microbial profiles between presence and absence of a certain factor/disease cannot be ruled out. Although we adopted strategies to deal with confounding, such as deriving a reference group free of medical conditions, remaining confounding factors are plausible. Having that in mind, our results highlight the usefulness of saliva as a tool to be investigated in order to improve diagnoses and monitoring of disease states.

In conclusion, this study indicates that age, periodontal status, sex, smoking, and certain medical conditions, named mental disorders and muscle and joint diseases, are associated with changes in the salivary microbiota.

## Supporting information

S1 File Microbial panel analysed by checkerboard DNA-DNA hybridization. (PDF)

S2 File STROBE Statement—Checklist of items that should be included in reports of *cross-sectional studies*. (DOC)

S3 File Study dataset. (XLSX)
