# Supplementary material for: Salivary microbial profiles in relation to age, periodontal, and systemic diseases
Source: PLoS One. 2018 Mar 14;13(3):e0189374. doi: 10.1371/journal.pone.0189374 (PMC5851536; doi:10.1371/journal.pone.0189374)
Supplement: S1 File — (PDF) [file pone.0189374.s001.pdf]

Supplemental file S1. Microbial panel analysed by checkerboard DNA-DNA hybridization.

**Specie**	**Collection**
Actinomyces israelii	ATCC 12102
Actinomyces naeslundii	ATCC 12104
Actinomyces oris	ATCC 43146
Actinomyces odontolyticus	ATCC 17929
Aggregatibacter actinomycetemcomitans	ATCC 43718, 29523
Campylobacter gracilis	ATCC 33236
Campylobacter rectus	ATCC 33238
Campylobacter showae	ATCC 51146
Capnocytophaga gingivalis	ATCC 33624
Capnocytophaga ochracea	ATCC 33596
Capnocytophaga sputigena	ATCC 33612
Eikenella corrodens	ATCC 23834
Eubacterium nodatum	ATCC 33099
Eubacterium saburreum	ATCC 33271
Fusobacterium nucleatum ss nucleatum	ATCC 25586
Fusobacterium nucleatum ss polymorphum	ATCC 10953
Fusobacterium nucleatum ss vincentii	ATCC 49256
Fusobacterium periodonticum	ATCC 33693
Gemella morbillorum	ATCC 27824
Helicobacter pylori	ATCC 43504
Leptotrichia buccalis	ATCC 14201
Neisseria mucosa	ATCC 19696
Parvimonas micra	ATCC 33270
Porphyromonas gingivalis	ATCC 33277
Prevotella intermedia	ATCC 25611
Prevotella melaninogenica	ATCC 25845
Prevotella nigrescens	ATCC 33563
Selenomonas noxia	ATCC 43541
Streptococcus anginosus	ATCC 33397
Streptococcus constellatus	ATCC 27823
Streptococcus intermedius	ATCC 27335
Streptococcus gordonii	ATCC 10558
Streptococcus mitis	ATCC 49456
Streptococcus oralis	ATCC 35037
Streptococcus mutans	ATCC 25175
Streptococcus salivarius	ATCC 27945
Streptococcus sanguinis	ATCC 10556
Tannerella forsythia	ATCC 43037
Treponema denticola	B1
Treponema socranskii	S1
Veillonella parvula	ATCC 10790

ATCC: American Type Culture Collection.
